# The impact of mental health recovery narratives on recipients experiencing mental health problems: Qualitative analysis and change model

**DOI:** 10.1371/journal.pone.0226201

**Published:** 2019-12-13

**Authors:** Stefan Rennick-Egglestone, Amy Ramsay, Rose McGranahan, Joy Llewellyn-Beardsley, Ada Hui, Kristian Pollock, Julie Repper, Caroline Yeo, Fiona Ng, James Roe, Steve Gillard, Graham Thornicroft, Susie Booth, Mike Slade

**Affiliations:** 1 School of Health Sciences, Institute of Mental Health, University of Nottingham, Nottingham, United Kingdom; 2 Health Service and Population Research Department, Institute of Psychiatry, Psychology and Neuroscience, King's College London, London, United Kingdom; 3 Unit of Social and Community Psychiatry, Blizard Institute, Barts and the London School of Medicine and Dentistry, Queen Mary University of London, London, United Kingdom; 4 School of Health Sciences, University of Nottingham, Nottingham, United Kingdom; 5 Implementing Recovery for Organisational Change (ImROC), Nottingham, United Kingdom; 6 National Institute for Health Research CLAHRC East Midlands, Institute of Mental Health, University of Nottingham, United Kingdom; 7 Population Health Research Institute, St. George's University of London, London, United Kingdom; 8 NEON Lived Experience Advisory Panel, Nottingham, United Kingdom; Universitat Luzern, SWITZERLAND

## Abstract

**Background:**

Mental health recovery narratives are stories of recovery from mental health problems. Narratives may impact in helpful and harmful ways on those who receive them. The objective of this paper is to develop a change model identifying the range of possible impacts and how they occur.

**Method:**

Semi-structured interviews were conducted with adults with experience of mental health problems and recovery (n = 77). Participants were asked to share a mental health recovery narrative and to describe the impact of other people’s recovery narratives on their own recovery. A change model was generated through iterative thematic analysis of transcripts.

**Results:**

Change is initiated when a recipient develops a connection to a narrator or to the events descripted in their narrative. Change is mediated by the recipient recognising experiences shared with the narrator, noticing the achievements or difficulties of the narrator, learning how recovery happens, or experiencing emotional release. Helpful outcomes of receiving recovery narratives are connectedness, validation, hope, empowerment, appreciation, reference shift and stigma reduction. Harmful outcomes are a sense of inadequacy, disconnection, pessimism and burden. Impact is positively moderated by the perceived authenticity of the narrative, and can be reduced if the recipient is experiencing a crisis.

**Conclusions:**

Interventions that incorporate the use of recovery narratives, such as peer support, anti-stigma campaigns and bibliotherapy, can use the change model to maximise benefit and minimise harms from narratives. Interventions should incorporate a diverse range of narratives available through different mediums to enable a range of recipients to connect with and benefit from this material. Service providers using recovery narratives should preserve authenticity so as to maximise impact, for example by avoiding excessive editing.

## Introduction

Mental health recovery narratives have been defined as first-person lived experience accounts of recovery from mental health problems, which refer to events or actions over a period of time, and which include elements of adversity or struggle, and also self-defined strengths, successes, or survival [1]. They are referred to as recovery narratives in this paper, whilst recognising that this term is used elsewhere in healthcare research and practice, e.g. in narratives of recovery after a stroke [2]. In keeping with prior research into the therapeutic use of narrative [3], we refer to the person telling the story as the **narrator**, and the person reading, watching or listening to the story as the **recipient**. Our choice of the term recipient has also been informed by reception theory, as articulated in media and cultural studies. This presents an understanding of reception as an active process of “decoding” messages generated by others, which is shaped by a range of personal and societal factors, and which in turn might influence how experiences or events are encoded into future messages [4]. In common with other forms of health narratives [5], recent research has shown that the process of receiving recovery narratives can make a short- and long-term change in recipients, and that this change can be both helpful and harmful [6, 7].

Recovery narratives can be shared live, as part of social interactions with others. They can also be presented in recorded form, most frequently as invariant text, audio, video [6], or visual artworks [8]. Informal peer support, involving interactions between individuals with similar experiences of health problems, is one example of a naturally-occurring relationship in which live recovery narratives can be narrated and received. Informal peer support can take place in-person [9] or on-line [10]. In this century a new employment role of peer support worker or peer specialist has emerged in mental health systems internationally [11], which involves employing people in roles for which the explicit use of personal experience of mental health problems and recovery is a requirement. Intentional peer support has an empirical evidence base [12] and is being implemented globally [13]. A USA national survey has identified helping others through the narrating of mental health recovery narratives as a feature of the work of peer specialists [14]. Peer support workers can create change through mechanisms such as role-modelling personal recovery [15]. The growth of peer support work means that an increasing number of people living with mental health problems have access to recovery narratives shared as part of a supportive relationship [16].

Recorded recovery narratives have been published in forms such as printed books [17–19] and health-service booklets [20, 21], which typically present either a single narrative or a curated collection of narratives from a range of contributors. Collections are often organised around recognisable mental health themes such as recovery from psychosis [18, 19]. Printed material is sometimes produced with an explicit editorial or narratorial intent of creating health-related benefits for others [22], such as providing a more positive outlook on a particular mental health condition to patients and carers [18], reducing stigma around a condition [18] and making recovery narratives available to service users [20, 21]. Books of recovery narratives are already being integrated as a resource in a range of mental health interventions [23], including the incorporation of autobiographies into the supportive environment of psychotherapy sessions [24, 25].

Recorded recovery narratives are available in ever-increasing numbers on-line. Like books, these can be organised into collections, often presented through bespoke web-sites. Examples include “What Recovery Means to Me”, published by the Scottish Recovery Network https://www.scottishrecovery.net/, and “Personal Stories”, published by Here to Help http://www.heretohelp.bc.ca/personal-stories. On-line collections can include a mixture of recovery narratives and narratives where recovery is sometimes present but not the primary focus, such as in the Schizophrenia Oral Histories Project https://schizophreniaoralhistories.com. Recovery narratives can also be shared individually, through digital media hosting services such as YouTube. Forms include recordings of public presentations [26] and first-person videos created specifically for sharing on-line https://www.youtube.com/watch?v=OCgREO2IJwQ.

Narrators of recovery narratives may choose to tell their story for a range of reasons, and may experience a range of benefits. The construction of alternative personal narratives to re-story experiences, for example to foster growth after trauma, is a central process in narrative therapy [27]. Constructing a narrative to share with others has been shown to have health benefits for the narrator across repeated international studies [28]. Developing and sharing a carefully-considered recovery narrative through a “Telling My Story” course in a Recovery College—where participants are supported to consider which aspects of their story they feel comfortable to share [29]—can provide an enhanced sense of self for a narrator [30]. Recovery narratives can facilitate a dialogue between clinicians and clients [31], and narrators of recovery narratives can be motivated by a desire to send messages of “hope, courage and survival” (p. 68) to others [32]. Both recorded and live recovery narratives have been used as an effective resource in anti-stigma campaigns [33] [34], and contributing to stigma-reduction might be a motivation for a narrator [35]. Activist groups have raised concerns that recovery narratives might be “co-opted”, e.g. used for purposes other than those intended by the narrator, and have argued that co-option can cause harm to narrators and others by sustaining harmful structures such as poorly functioning health services or economic arrangements [36].

The process of receiving a narrative can create helpful change in recipients. Receiving a recovery narrative with elements that resonate with personal experience can provide personal inspiration [30], increase empathy and understanding [37], validate difficult personal experiences [38], or provide alternative forms of companionship at times of social isolation [39]. However, recovery narratives can also contribute to recipient distress, e.g. if the recipient feels angry or “out of place” through a perception that they have experienced greater hardship than a narrator [30]. A systematic review [6] of empirical research into the impact of mental health recovery narratives on recipients identified six broad categories of impact: Connectedness (to the narrator and others); Understanding of recovery; Reduction in stigma; Validation of personal experience; Emotional responses; and Behavioural responses. Impact was moderated by characteristics of the recipient, the context of the narrative, and the characteristics of the narrator or narrative. Diagnostically-specific harms were identified in a sub-group analysis of publications specific to eating disorders [39–41], including emulation of eating disorder behaviours described by a narrator [41] and triggering of previously-experienced behaviours [41]. This is consistent with research which demonstrates potentiation of harmful behaviours by receiving on-line material describing harmful behaviours [42].

The systematic review described above only included five studies and concluded by identifying a knowledge gap around the impact of recovery narratives. Four included studies were specific to particular diagnoses [38–41]. Three studies used methods in which researchers provided recovery narratives [38, 40, 41]. Three featured a time of impact assessment which was three months or less [38, 40, 41]. One study only included eight participants [30]. Included studies showed a collective gender bias (178 F, 23 M), and all but one provided insufficient information to identify other demographic biases. All were conducted in high-income countries. Collectively, this means that current work does not provide sufficient information to understand impact as it occurs naturalistically, through interaction with live or recorded recovery narratives. Although the forms of impact identified by this systematic review provide some insight into how receiving a recover narrative creates change in an individual, the review did not attempt to produce a formal model for how and what change occurs during the process of reception, and no included paper presented such a model.

The study reported on in this paper has taken place as part of the Narrative Experiences ONline (NEON) study (http://www.researchintorecovery.com/neon), which aims to evaluate the use of recorded recovery narratives as a mental health intervention. UK Medical Research Council guidance [23] recommends the modelling of how health-related change occurs during the development and evaluation of interventions. The modelling of change caused by receiving recovery narratives is particularly important when prior research has identified that harmful impact can occur, and since the ever-growing public availability of recovery narratives, and in particular their use in mass-scale anti-stigma campaigns, means that individuals might encounter them outside of the support of a therapeutic environment [6].

To support the safe and effective use of mental health recovery narratives in complex mental health interventions, the aim of this study was to develop a preliminary model describing how receiving live and recorded recovery narratives might create health-related change, which could then be validated and refined through future studies.

## Methods

A preliminary change model was developed through the iterative thematic analysis of semi-structured interviews, in which participants were asked to share a recovery narrative, and were interviewed about the impact of recovery narratives (amongst other topics). Research was undertaken between March and August 2018. Ethical Committee approval was obtained in advance (Nottingham 2 REC 17/EM/0401). All participants provided written informed consent. Findings will inform a future trial (ISRCTN11152837).

### Participants

Participants were recruited to four groups. Inclusion criteria across all four groups were: aged 18 years and over; able to give written informed consent; fluent in English and willing to discuss their personal experiences, including in the form of a recovery narrative. Inclusion criteria for each group: *Outside the system*: lifetime experience of psychosis, and no use of secondary mental health services for five years. *Black and minority ethnic*: identify as a member of a Black and/or Minority Ethnic (BME) population, and are using or have used mental health services. *Under-served*: experience of mental health problems, and with either no or difficult experiences with mental health services. *Peer worker*: experience of working in statutory or voluntary roles where lived experience is used as a tool for engagement with mental health service users. The *peer workers* group was included due to a likelihood that members would have an everyday familiarity with sharing and receiving recovery narratives. *Outside the system*, *BME* and *Under-served* were included because informal peer support, including the sharing of recovery narratives, is common in underserved or excluded groups [43], and because under-served groups are currently under-represented in research around recovery narratives [1]

### Setting

All participants were recruited in England. *Outside the System* was recruited through primary care services, Hearing Voices Networks and online support groups. *BME* was recruited through voluntary groups, recovery colleges and secondary mental health services. Prior research has identified consistent patterns of mental health service under-provision for people with multiple and complex health and social care needs (MCN) [44], from rural communities [45] and from lesbian, gay, bisexual or trans (LGBT) communities [46], and hence *Under-served* was recruited through community networks, voluntary sector organisations or other forms of provision serving these. *Peer worker* was recruited through voluntary groups and secondary mental health services.

### Procedures

Most potential participants were made aware of the study through display of posters in relevant settings, through direct approach by recovery college staff or by statutory or voluntary health or social care providers, through attendance of research team members at meetings or conferences held by relevant special interest group, by social media or website postings, by word of mouth and by snowball sampling. Some participants in *Outside the System* were recruited by invitation letters sent as a result of scanning primary care records for potentially eligible participants. All recruitment material indicated that participants would be asked to share a recovery narrative. This was due to the contested nature of this term [47], and therefore formed an early part of our informed consent procedures. One research participant was a study team member. For the safety of both the researcher and the participant, their interview was conducted by a researcher working at a different site, who had explicitly confirmed that they were comfortable conducting the interview.

Potential participants were asked to contact the research team, and each had a discussion with a researcher by phone or in person. At this, the researcher assessed eligibility, explained that the study involved sharing a recovery narrative, provided a Participant Information Sheet (PIS) (in print or electronic form), and provided an opportunity to ask questions about the study. Potential participants were asked to confirm their interest in participation once they had absorbed the PIS and consulted with others if needed. Once a potential participant had confirmed their intention to take part, an interview date and location was confirmed. This was either in a community or health service venue, depending upon the preference of the participant. Consent was taken in writing at the start of the interview, using a printed Informed Consent Form.

Semi-structured interviews were conducted by four researchers with backgrounds in advocacy, health psychology, public health and sociology. Training for interviews was provided by the NEON study Lived Experience Advisory Panel (LEAP), who role-played interview participation, and provided feedback on the effectiveness and personal impact of interview procedures. LEAP members also contributed to topic guide development. The topic guide was developed iteratively by the research team as our understanding of how to best conduct interviews developed. The topic guide produced at the end of this iterative process is included in S1 Text.

Interviews were audio-recorded, transcribed and anonymised, and participants completed an anonymous demographics questionnaire whilst attending the interview. Each interview followed a two-part topic guide. In part A, participants were asked an open ended question designed to elicit a recovery narrative [48]. Prompts from the interviewer were minimised [49] to allow the narrator scope to tell their story in their own way, but were introduced if needed. In part B, participants were questioned about the impact of recovery narratives on themselves and others, amongst other questions.

### Analysis

A preliminary change model was developed through an iterative thematic analysis of collected transcripts. This began with an inductive thematic analysis of all BME transcripts, using NVIVO 11 for data organisation and coding. A preliminary coding framework was first generated by a single researcher (AR). This identified (1) forms of impact and (2) moderators of impact. *Moderators* was included as a pre-defined superordinate category, informed by the results of the prior systematic review [6], and by findings from audience reception theory which suggest recipients will interpret the same narratives differently depending on a range of factors, such as their cultural background or age [4]. Transcript text from both parts A and B was considered as in scope for this analysis; part A because field notes indicated that, in telling their recovery narrative, participants frequently referred directly to the impact of others’ recovery narratives on their own recovery, and part B due to the specific focus of questions asked by interviewers. In conducting this analysis, no separation was introduced between impact caused by live and by recorded recovery narratives.

The preliminary coding framework was considered in meetings of a broader analysis team, incorporating three researchers who had conducted the interviews (AR, JB and RM), and also three analysts with expertise in healthcare technology, qualitative research, and recovery research and clinical psychology (SRE, KP and MS). Several team members also had lived experience of mental ill-health and recovery, enhancing the role of lived experience in data analysis [50]. Code names and definitions were refined, and forms of impact were classified as helpful or harmful to recovery, led by participant accounts of these. The preliminary coding framework was refined by applying it to the remaining transcripts, and the refined coding framework is presented in S1 Table. Coding was undertaken by four researchers (AR, JB, AH and RM), each allocated 25% of the remaining transcripts. Second coding of 20% of all transcripts was conducted by SRE, to verify that coded material related to the impact of recovery narratives, with transcripts selected for maximum number of coded instances of impact.

To produce a change model, narrative summaries of each NVIVO node were first produced, to enable easy comprehension of the full range of material collected. Working from these narrative summaries, forms of impact were partitioned into the superordinate categories of mechanisms, mediators, moderators, helpful outcomes, and harmful outcomes. Impact was classified as an outcome if it represented a change to cognition, emotion or behaviour. It was classified as a mechanism if it caused this change. It was classified as a mediator if it was part of a pathway that enacted that change, and depended on a mechanism to occur. It was classed as a moderator if it influenced the degree of change.

To support the work of the NEON study, a planned sub-group analysis was conducted of material identifying specific benefits of receiving recorded recovery narratives.

## Results

### Summary of participants

Sociodemographic and clinical characteristics of the 77 participants are shown in Table 1.

**Table 1:** Sociodemographic and clinical characteristics of participants (n = 77).

Characteristic	Total	Outside the system	Black and Minority Ethnic	Under-served	Peer workers
n (%)	*77 (100)*	*21 (27)*	*21 (27)*	*19 (25)*	*16 (21)*
**Gender** n (%)					
Female	42 (55)	14 (67)	11 (53)	8 (42)	9 (56)
Male	30 (39)	6 (29)	9 (43)	9 (47)	6 (38)
Other / prefer not to say	5 (6)	1 (5)	1 (5)	2 (11)	1 (6)
**Ethnicity** n (%)					
White British	44 (57)	12 (57)	0 (0)	18 (95)	14 (88)
Black British	5 (6)	2 (10)	3 (14)	0 (0)	0 (0)
Black African / Caribbean	4 (5)	1 (5)	3 (14)	0 (0)	0 (0)
White Other	5 (6)	2 (10)	1 (5)	0 (0)	2 (13)
White and Black African / Caribbean	4 (5)	0 (0)	4 (19)	0 (0)	0 (0)
Asian / Mixed white Asian	4 (5)	0 (0)	4 (19)	0 (0)	0 (0)
Other	5 (6)	2 (10)	3 (14)	0 (0)	0 (0)
Prefer not to say	6 (8)	2 (10)	3 (14)	1 (5)	0 (0)
**Age** (years)					
18–25	4 (5)	0 (0)	0 (0)	3 (16)	1 (6)
25–34	16 (21)	3 (14)	6 (29)	4 (21)	3 (19)
35–44	16 (21)	5 (24)	4 (19)	4 (21)	3 (19)
45–54	30 (39)	8 (38)	9 (43)	6 (32)	7 (43)
55+	5 (6)	4 (19)	0 (0)	0 (0)	1 (6)
Prefer not to say	6 (8)	1 (5)	2 (10)	2 (11)	1 (6)
**Sexual orientation**					
Heterosexual	49 (64)	15 (71)	14 (67)	6 (32)	14 (88)
LGBT+	18 (23)	3 (14)	4 (19)	9 (47)	2 (13)
Prefer not to say	10 (13)	3 (14)	3 (14)	4 (21)	0 (0)
**Primary diagnosis**					
Schizophrenia or other psychosis	11 (14)	5 (24)	4 (19)	2 (11)	0 (0)
Bipolar disorder	16 (21)	8 (38)	1 (5)	3 (16)	4 (25)
Mood disorder, e.g. anxiety, depression, dysthymia	15 (19)	1 (5)	4 (19)	4 (21)	6 (38)
Other, e.g. ADHD, personality disorder, substance abuse, autism	7 (9)	0 (0)	2 (10)	3 (16)	2 (13)
Prefer not to say	28 (36)	7 (33)	10 (48)	7 (37)	4 (25)

### Outcomes

Seven helpful outcomes of receiving recovery narratives were identified, summarised in Table 2.

**Table 2:** Helpful outcomes, illustrated by examples coded in transcripts.

Outcome	Example text
**1. Connectedness**	
Feeling less alone	Well I just think it was you know hearing people's stories and kind of feeling that my God like I am not alone [C006: Female, 25–34, Under-served, lives in a rural location]
Feeling more like a community member	A weird part of my recovery has definitely been connecting with black women, not just about their trauma but I kind of became more connected with listening to black women's stories [B024: Female, 25–34, BME]
Feeling more connected to specific individuals	there's a girl I know … She was abused…. people just living their everyday lives wouldn't even think about it … I am pretty close to her, anyway you know I look out for her. [C012: Male, 35–44, Under-served, multiple and complex needs]
**2. Validation**	
Normalising personal experiences	going to those festivals and meeting other people who had been through similar stories to me, that was pretty amazing … there is validation everywhere … I found that incredibly helpful. [A010: Female, 35–44, Outside the system]
Reconceptualising mental health problems as a collective experience	Listening to someone else can give you validation in your experiences that actually it isn’t you, that this is a collective experience. [B020: Female, BME]
**3. Hope**	
Feeling more hopeful about what achievements are possible	there are other people that have been through what you've been through and come out the other side, and are doing amazing things… just knowing … it helps a massive lot. [B021: Female, 35–44, BME]
Feeling more optimistic about human nature	I think it is to find out they are such lovely people inside, like deep inside they are just normal people first of all … such a nice soul, they are wonderful, lovely people … very very inspiring. [A009: Female, 35–44, Outside the system]
**4. Empowerment**	
Enhanced ability to share personal narratives	when you see somebody talk about things, you think ‘wow’ … that gives you confidence as well … It gives you that, ‘OK, I want to talk about it, I want to talk about my experience as well’. [B016: Male, 25–34, BME]
Enhanced belief that systems can be challenged	she was taking … the County Council to the Supreme Court …she had been placed into a dangerous foster home … I thought there was no way that she will win, because if she wins that is going to have massive implications, but she did, she won [C019: Under-served, multiple and complex needs]
Enhanced ability to make personal change	And something about just talking to her that night … I just thought I'm not taking the tablet … I didn't have a clear idea that I was going to stop but I didn't restart [Female, 35–44, Outside the system]
**5. Appreciation**	
Enhanced appreciation of positive elements in the recipient’s life	I've caught myself plenty of times thinking … my life hasn't got as bad as his yet, I'm all right … selfish of me I thought [C007: Male, 45–54, Under-served, multiple and complex needs]
Enhanced appreciation of the challenges others experience	I have heard stories of other people with abuse issues … I thought yeah mine's bad, but I was pretty humbled. … I am very humbled to hear other people's stories [D016: Female, 25–34, Peer worker]
**6. Reference shift**	I kind of thought that that was the best I could ever hope for … I kind of really internalized my label of I am an anorexic … it was seeing the people who were talking about their lived experience with lots and lots of confidence and taking lots of responsibility … which kind of opened my eyes to like gosh maybe I am more than my label … maybe I could do more than just function or survive, maybe I could thrive [D014: Female, 45–54, Peer worker]
**7. Stigma reduction**	
Reduction in shame	I saw so many common themes running throughout the stories, many, many people said they were sensitive and felt different as a child … that was really helpful in healing my own shame [A011: Female, 35–44, Outside the system]

Four harmful outcomes were identified, summarised in Table 3.

**Table 3:** Harmful outcomes, illustrated by examples coded in transcripts.

Outcome	Example text
**1. Inadequacy**	
Others have made a better recovery	oh they’ve done so much better than me, look they’ve had a—you know—a book published, or they’ve done this or that or—they’re on a Band 6 or something, [C003: Male, 25–34, Under-served, LGBT]
**2. Disconnection**	
From others who have experienced recovery	Other people say oh well I did this … and it made me feel better. It’s like oh fine, that's great for you mate but it doesn't touch the sides because I am hollowed out and empty and in pain [D004: Male, Peer worker]
From narrators experiencing less distress	Oh, people will talk about uh what can I say … Yeah, so one lady was talking about a pet, she lost her pet or something, and I was sitting there thinking, oh what are you bringing that up for? [C003: Male, 25–34, Under-served, LGBT]
**3. Pessimism**	
About how much recovery is possible	So yeah, it is not nice to hear that people haven't come out the other and gone back to themselves [A014: Female, 25–34, Outside the system]
About the problems of society	What is in the ether, what is in the world that is creating these people to have these stories? [B020: Female, BME]
About the value of sharing narratives	the pessimist in me says, why share your story? What’s the point in it? And I have a real issue with the notion of Survivor … And someone’s stood up and given a really heartfelt story, and people clap. I just think, how patronising is that? [B020: Female, BME]
**4. Burden**	I also did notice the days after I would feel really low, because I would then process what they have said and I would feel so much for them … it would be really overwhelming [A002: Female, 25–34, Outside the system]

Analysis of parts A and B of the transcripts yielded a rich body of evidence about the outcomes of receiving recovery narratives, and hence further examples of text coded against each sub-theme are provided in S2 Table.

Outcomes could occur in parallel, as in the following quote describing the helpful outcomes of connectedness and validation:

*when you hear someone’s stories and you meet people with lived experience you feel part of that*, *like a community*, *a little community and yeah the validation that’s inspiring*

*[D005*: *Male*, *45–54*, *Peer worker]*

Reference shift and burden did not have any subtypes coded. Reference shift was a fundamental change in belief or understanding about the possibility of recovery, and how it might come about. Burden referred to a recipient feeling weighed down by the distress that they had encountered in others’ narratives.

### Mechanisms

Connection to the narrator and/or their narrative was identified as the single mechanism by which the process of recovery narratives making an impact was initiated. Connection was sometimes manifest as a perceived empathic connection to the narrator, as shown in the following quote, where it leads to appreciation:

*I feel … empathy … I don't feel everything*, *but if I feel even just a little bit of what they've gone through*, *it's incredible to me … it puts things in perspective I think*, *so it's not a pleasant feeling*, *but I think it's a valuable one to go through*, *because you can sort of see what it has really been like for someone*

*[D003*: *Male*, *18–24*, *Peer worker]*

More frequently, connection occurred through the recipient comparing themselves to the narrator or their narrative, and finding some form of match or common ground. Comparisons could be against shared characteristics (gender, ethnicity, profession, mental health diagnosis, economic situation, social status, severity of distress, frame of reference, and degree of recovery). They could also be through the recipient comparing features of their own current internal narrative to features of the narrative they had received. Comparisons incorporating recovery events, health system experiences (positive and negative), and difficult family interactions were observed.

The following quote illustrates a comparison on gender and mental health diagnosis::

*We did some group work with some women … about psychosis*. *And I started to think*, *you know*, *I’m not really on my own*, *you know*. *This*, *it is a very lonely world but I’m not on my own*.

*[B006*: *Female*, *45–54*, *BME]*

Whilst the following illustrates a comparison on profession:

… *yeah reading our mutual friend's book was really good*, *I think just to see … here is someone that had also worked in the same circle as me and had gone through it and I think that really helped me knowing that there's other people in my field that had experienced it*.

*[A018*: *Female*, *25–34*, *Outside the system]*

When making a comparison on degree of recovery, a recurrent theme was that a mismatch disrupted connection to the narrator, and hence impact of the narrative:

*Listening to people's problems on why*, *mental health*, *how they've recovered and all that lot*, *I don't want to know because I might be at that stage where I'm really weak*

*[C004*: *Female*, *35–44*, *Under-served*, *lived in a rural area]*

The following quote illustrates a comparison on a recovery event, in the form of a spiritual emergence experience encountered by both recipient and narrator.

*meeting other people who had been through similar stories to me*, *that was pretty amazing … this guy who I met who helped me to escape from hospital when I was sectioned*, *he had had a spiritual emergence experience when he was in his early 20's … it sounded similar to mine*

*[A010*: *Female*, *35–44*, *Outside the system]*

If a comparison was made to a narrator who was very different, then this could negate impact.

*Or if it’s well*, *I’ve just got nothing in common with that*. *Absolutely nothing in common*, *I’ve not had that education*, *I’m not the same gender*, *I’m not the same sexuality as them*

*[D002*: *Female*, *55–64*, *Peer worker]*

### Moderators

Two factors moderating impact were identified: recipient in crisis and narrative authenticity.

#### Moderator 1: Recipient in crisis

Experiencing a crisis could reduce the help that could come from recovery narratives.

*Um*, *and I think if you was- if you was really poorly it probably wouldn't be very beneficial to hear somebody else’s stories too much because um you can't understand your own at that time*

*[C003*: *Male*, *25–34*, *Under-served*, *LGBT]*

Sharing or receiving narratives during a period of crisis appeared to be a cause of harm for some:

*sometimes*, *when I was in hospital and we had times when had to share stories and hear people's stories …somebody told you something and they were so explicit of how it happened to them*, *you start to go through those emotions yourself and start to hear what they were hearing and experience what they were experiencing*. *So sometimes it can be*, *if there is too much going on*. *If you are trying to deal with your own and you're*, *you know you're asked to share or you are hearing other people's stories it can affect you*.

*[A013*: *Female*, *35–44*, *Outside the system]*

#### Moderator 2: Narrative authenticity

A recovery narrative perceived as authentic appeared to have a greater impact, and a narrative perceived as inauthentic a lesser impact. Perceptions of authenticity were increased if narrator told the whole of their journey, without apparent editing of difficulties, summarised as:

*when people are genuine and transparent in what they say … genuinely say that’s where they were*, *to where they are now*, *you know*, *that’s the sort of thing I like but it has to be authentic*.

*[B002*: *Male*, *45–54*, *BME]*

Perceptions of authenticity were decreased if the narrative appeared embellished or inconsistent:

*I think more than anything it's how they change the stories when they've been*, *when they tell them*. *They'll tell them and then half an hour later they'll forget what they've said and there will be something different about it*

*[C010*: *Male*, *35–44*, *Under-served*, *multiple and complex needs]*

They were also decreased if the purpose of the narrative was to promote a health service function:

It’s often about their experiences of a particular service, so t’s usually organised by that service where they get people that have used it to come and say how great the service was and about their experience is of it and that they may well have re-built their lives, but it’s just the way that its presented and I just sort of sit there thinking oh no not another person who has been wheeled on

[D011: Female, 45–54, Peer worker]

### Mediators

Five mediators were identified. Each could be on a pathway to multiple outcomes. Mediators are not intended to be mutually exclusive—a recipient might, for example, recognise shared experiences, notice narrator achievements and notice narrator difficulties, all through receiving the same narrative.

#### Mediator 1: Recognising shared experiences

The outcomes of validation, connection, and stigma reduction could all be produced through a recipient recognising experienced shared with a narrator.

In the outcome of validation, recognising shared experiences helped a recipient to learn that their own experiences were more normal than they had thought, in that they had been experienced by many others:

*gathering the stories for <anon book> has been immensely helpful for me*. *Because it helped*, *yeah it helped validate my experience as I saw so many common themes running throughout the stories*, *many*, *many people said they were sensitive and felt different as a child*

*[A011*: *Female*, *35–44*, *Outside the system]*

In the outcome of connection, recognising shared experiences resulted in the recipient feeling more connected to others, or to the world around them.

*we're still in contact because we really bonded over shared experiences which we'll never have with other people*, *no-one will understand some of the things that we go through*

*[D014*: *Female*, *45–54*, *Peer worker]*

Recognising shared experiences could lead to the helpful outcome of stigma reduction when this helped recipients to let go of a sense of shame that they had held within them.

#### Mediator 2: Noticing narrator achievements

Hope, empowerment, inadequacy and disconnection could all be produced through noticing the elements of recovery narratives that related to success, strengths or survival, and which served to communicate the achievements of the narrator.

For some recipients, noticing narrator achievements produced hope, i.e. an enhanced belief that such achievements might be possible for the recipient:

*I was- that kind of made me think to myself yeah hearing their story*, *I hope I can be like that*. *You know as long as to be able to share it because by them doing that has given me a lot of hope*

*[C003*: *Male*, *25–34*, *Under-served*, *LGBT]*

For others it led to the outcome of empowerment, which was a more immediate and enhanced ability to start working towards achievements or other forms of change.

*hearing from other people’s stories made me brave*. *That’s the best way to talk about it*. *Made me brave to make changes for the better*. *…*

*[B016*: *Male*, *25–34*, *BME]*

The harmful outcome of inadequacy could be produced when the recipient felt that they could not achieve what the narrator had achieved, as in the example in Table 2. The harmful outcome of disconnection could be produced when noticing narrator achievements caused a recipient to distance themselves from the narrator:

*he does go on about his story so sometimes he will dominate the conversation to tell his story because he enjoys telling his story … it's not particularly helpful sometimes when they go on like evangelical about their recovery journey*.

*[B005*: *Female*, *35–44*, *BME]*

#### Mediator 3: Noticing narrator difficulties

Appreciation, disconnection, pessimism and burden could all be produced by noticing the elements of recovery narratives that related to difficulties.

Pessimism came about when noticing difficulties made a recipient feel less optimistic about prospects for their own recovery or of others:

*I guess some of the stuff that kind of focuses on how hard it is to get support can potentially make me feel like*, *it's hopeless*, *why am I banging my head against a brick wall*, *keep going back to the GP*, *when this is something that you can see across the board is just shit*, *provision is shit*, *provision is not where it needs to be*. *It's kind of disheartening*,

*[C014*: *Female*, *25–34*, *Under-served*, *LGBT]*

Disconnection came about when a recipient perceived that their own difficulties were greater or more distressing than those of the narrator:

*Oh*, *people will talk about uh what can I say* …*Yeah*, *so one lady was talking about a pet*, *she lost her pet or something*, *and I was sitting there thinking*, *oh what are you bringing that up for*?

*[C003*: *Male*, *25–34*, *Under-served*, *LGBT]*

Burden was produced through repeatedly noticing narrator difficulties, especially for those recipients who described themselves as empathic. This could be produced through receiving recovery narratives, as in the example in Table 2, but was more frequently described in relation to receiving narratives purely of distress, with no recovery element present, as for the following participant who had taken part in a group support session:

*Yeah*, *when they don't see any bright side*, *when it's all doom and gloom and … there's nothing that's enlightening*, *there's nothing that will help anybody else go through it*, *so sometimes you come away and you feel quite depressed because there's- you're left in a hopeless state then*.

*[A016*: *Female*, *45–54*, *Outside the system]*

#### Mediator 4: Learning how recovery happens

Hope, empowerment and reference shift could all be produced by learning from narrators about how recovery happened for them. Learning about how recovery happened encompassed learning that recovery is possible:

*It gives me that boost*, *it's like you know you can survive if you get the right help and you use the right tools and you educate yourself enough you can be your own you know saviour*

*[C006*: *Female*, *25–34*, *Under-served*, *lives in a rural location]*

It also encompassed learning about alternative conceptualisations of mental health problems which had helped narrators, learning about recovery strategies, and learning about possible future barriers to recovery,

*I'd read an account by this woman called Melissa Gunasena and she*'*d written an account of her experiences of kind of psychosis and she'd also understood them in a very spiritual way*

*[A010*: *Female*, *35–44*, *Outside the system]*

*you can share your own stories and share other people’s stories … you can pass that information to them or you can get advice from them as to how they got through it as well*.

*[B007*: *Male*, *45–54*, *BME]*

*It gives you insight into what might be coming up for you*. *So someone might share their story … and you can recommend or you know what pitfalls are coming ahead*

[B020: Female, BME]

#### Mediator 5: Experiencing emotional release

Hope, reference shift and connectedness could all be produced through a recipient experiencing emotional release, which could be experienced as rapid, powerful and visceral:

… *(long pause) and it just felt like someone had punched me in the stomach and just kind of sat there crying for ages*

*[A006*: *Male*, *35–44*, *Outside the system]*

*And that was amazing and every story I read*, *it resonated so much with me*, *how I was after <anon daughter 1> and <anon daughter 2> and it was just so emotional and it was just floods of tears*

*[D005*: *Male*, *45–54*, *Peer worker]*

Narratives sometimes brought a recipient close to an emotional release. This was sometimes referred to as “bringing stuff up”, as in the following example of emotional release through an encounter with a narrative of distress:

*Yeah I think so*, *I think hearing about others peoples traumas is sometimes quite difficult like*, *sometimes because I can relate to it and it’s painful and it brings stuff up*

*[C002*: *Female*, *18–24*, *Under-served*, *lives in a rural location]*

This form of partial emotional release could produce both connection and burden, the latter apparently through painful emotions sitting just under the surface.

### Change model

**Fig 1:**
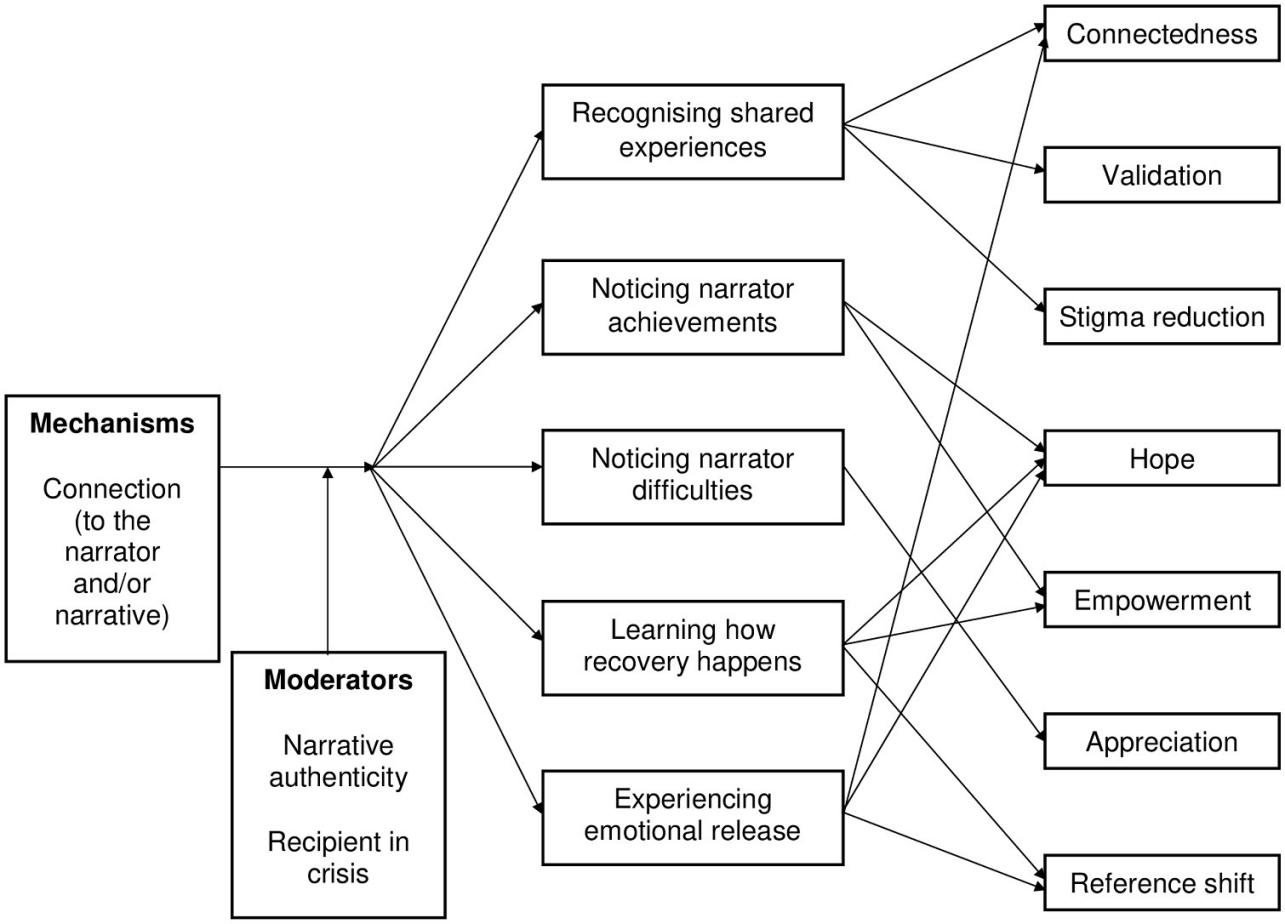
Change model identifying how helpful outcomes occur.

**Fig 2:**
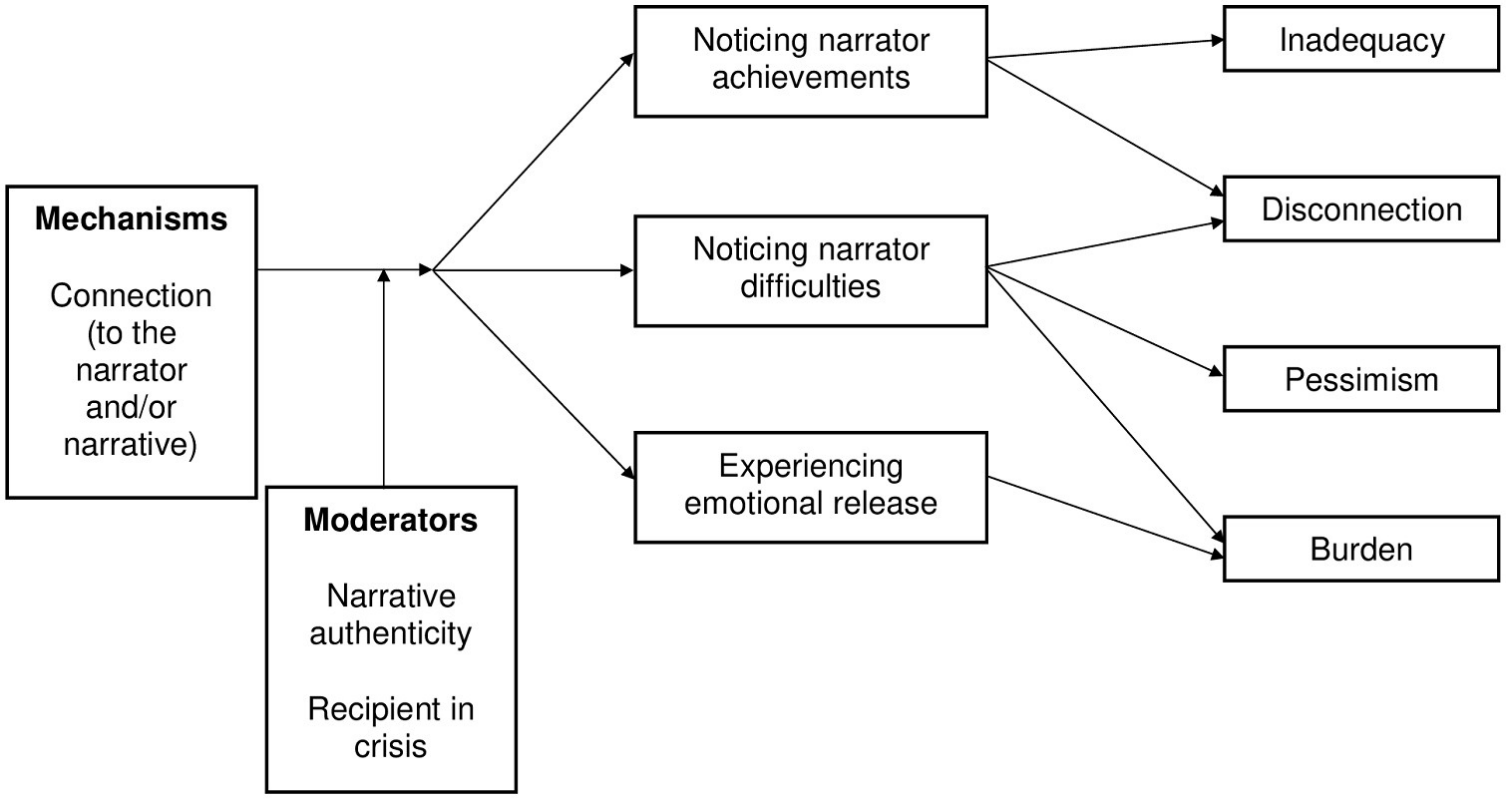
Change model identifying how harmful outcomes occur.

The change model, comprised of the concepts presented above, is summarised in Figs 1 and 2, where it has been separated into two components for clarity of presentation. Fig 1 presents a model explaining how helpful outcomes occurred in transcript data.

Fig 2 presents a model explaining how harmful outcomes occurred.

The relationship between some of the elements in the change model is demonstrated in the following longer excerpt from a transcript.

*I didn't really go on antidepressants*, *I just took them for three weeks … I felt so low … I was in this peer support group which is an on-line group and there was another woman in the group*, *it was just the two of us that week and just hearing her talk and listen to me and share her experiences which were so similar because she'd … had a real sense that she … wanted to go down a really different route with her acting and it was more- It was more kind of self-driven and less about pleasing other people and she'd felt really strongly that was the way she needed to go*, *I felt that's my story too*, *that's how I felt*, *I need to- I don't know just find my own power and not try to please others and be something I'm not*. *And something about just talking to her that night*, *I just thought the next day*, *I just thought I'm not taking the tablet*, *I'm just not taking it and the next day*, *I was*, *I'm not going to take it and I didn't have a clear idea that I was going to stop but I didn't restart*.

*[A010*: *Female*, *35–44*, *Outside the system]*.

Here, helpful outcomes are empowerment and reference shift. Empowerment is in the form of an enhanced ability to make choices over medication usage. Reference shift can be seen in an overnight change in understanding of how recovery might happen or what recovery might look like. Change has happened because the recipient has developed a connection to both the narrator and their narrative, by identifying a match on frame of reference and similarity of experience. Effects are mediated by the recipient noticing narrator achievements and learning about how recovery happened, which in this case involved making profound personal changes that benefited recovery. These changes may have been moderated by the authenticity of the narrative, although the latter is not explicitly stated in this fragment.

### Benefits of recorded recovery narratives

Three types of benefit specific to receiving recorded recovery narratives were identified through a sub-group analysis of transcript material. These benefits are summarised in Table 4.

**Table 4:** Benefits of recorded recovery narratives, illustrated by examples coded in transcripts.

Benefit	Examples
1. Obtaining access to narrators not available in everyday life	I didn't have like a mentor, I didn't have um somebody to look up to in recovery; I had to seek that out but knowing that that was there, [C004: Female, 35–44, Under-served, lives in a rural location] Mostly books … if you've been through whatever, you actually, actively are searching for you know some correlating or like some- somebody's experiences to sort of match yours. … There must be other people who have had this. [A008: Female, 45–54, Outside the system]
2. Control over when and how to access a narrative	because you can dip in and out of it easier. And you choose to pick that up when you are ready to talk about it, you can log off, you can put it down whenever you need to [C014: Female, 25–34, Under-served, LGBT]
3. Lack of social interaction burden around a narrative	And there is less of an expectation on you in that circumstance than there is when you are kind of with someone in person [C014: Female, 25–34, Under-served, LGBT]

In the table above, participant C004 is describing her escape from a sustained period of social isolation, which was assisted through encountering recovery stories in books, on the television and on the Web. Participant A008 is describing a turning point in her own recovery which was caused by reading recovery narratives in books. Participant C014 had previously described the negative impact of other’s mental health narratives on herself when she was not ready to hear them, and hence the benefits of encountering recovery narratives presented in forms over which she had control.

## Discussion

A model describing how mental health recovery narratives can create health-related change was developed through iterative thematic analysis of semi-structured interviews with 77 participants. In the model, change is initiated when a recipient develops a connection to a narrator and/or their narrative, and is mediated by the recipient recognising shared experiences, noticing narrator achievements, noticing narrator difficulties, learning how recovery happens or experiencing emotional release. Helpful outcomes of receiving recovery narratives are connectedness, validation, hope and optimism, empowerment, appreciation, reference shift and stigma reduction. Harmful outcomes are inadequacy, disconnection, pessimism and burden. Impact is positively moderated by the perceived authenticity of the narrative, and can be reduced if the recipient is experiencing a crisis. A sub-group analysis identified three specific benefits to a recipient of receiving recorded recovery narratives: obtaining access to narrators not available in everyday life, control over when and how to access a narrative, and lack of social interaction burden.

### Relationship to prior research

This paper can be situated within a small body of existing work examining the impact of mental health recovery narratives on recipients. Ours is the first to present a formal change model for the impact of recovery narratives, but other works have included some discussion of health-related change and how it occurs. The mechanism of connection described in our change model appears to extend a process labelled as “self to other comparison”, described in outline in [30], and capable of producing both positive and negative change. Our analysis identifies a range of specific characteristics, events or experiences utilised in self-to-other comparisons, which are not described in [30], and hence our analysis enhances an understanding of this process. The mechanism of connection described in our analysis also incorporates a perception of empathic connection to the narrator, strongly present in some of our transcripts, and not included in [30].

Our analysis has identified a mediating role for learning about how recovery happens, and has also identified four sub-types: learning that recovery is possible, learning about alternative conceptualisations of mental health problems, learning about recovery strategies, and learning about possible future barriers to recovery. These sub-types complement processes described as *solidifying existing knowledge*, and *learning new approaches to try*, documented in [38]. A role for recovery narratives in helping recipients learning about alternative conceptualisations of anorexia nervosa has been identified in [40]. However, experience of anorexia nervosa was not an inclusion criteria for participants, and impact was assessed on the same day as a recovery narrative was received, which means that the impact of this on the longer-term personal recovery of participants could not be examined.

A systematic review [6] has identified changes in behaviour as a category of impact. Our study locates behavioural changes summarised in this review, such as changes in adherence to medication programmes, within the more general outcome of empowerment, specifically the sub-theme of enhanced ability to make personal change. Harmful, diagnostically-specific changes to behaviour identified in prior work, such as triggering of prior eating disorder behaviours [41], were not present in our analysis, perhaps because only one participant listed an eating disorder as a primary diagnosis. Our study is the first to identify trans-diagnostic harms through receiving recovery narratives, in the form of the four harmful outcomes of inadequacy, disconnection, pessimism and burden.

The same systematic review positions empathy for a narrator as an initiator for strongly-felt emotions. Our work confirms this in two ways, by positioning empathy as part of the mechanism of connection, and by observing that noticing narrator difficulties could lead to burden for empathic recipients. Burden caused through repeatedly noticing narrator difficulties might be related to a broader body of work on vicarious trauma or secondary traumatisation in healthcare professionals, where the ability to feel empathy for others is known to be a key risk factor [51].

Our analysis is the first to identify a role for emotional release in mediating the impact of receiving recovery narratives. Trigger warnings are sometimes presented as a helpful tactic in material that might elicit strong emotional responses, particularly in those currently experiencing mental health distress [52]. They are frequently used in on-line websites and education material [53]. However, if a trigger warning leads a person to avoid a piece of media, then they may have precluded some of the helpful outcomes seen in our corpus. Furthermore, the empirical evidence in relation to psychosis is clear that post-traumatic growth is both possible and common [54]. Research is needed into the relationship between receiving narratives which cause short-term emotional distress and longer-term post-traumatic growth.

Our analysis has identified perceived authenticity as a moderator for the impact of mental health recovery narratives. Authenticity might be related to the broader and more heavily researched notion of *credibility* as it relates to health information seeking. This has been defined in a systematic review as “the end result of series of judgments people apply during their online search processes”, with sources perceived as more credible being more influential [55]. It might also be related to the concept of “perceived realism”, which has been considered in the study of health communications, where it has been identified as a moderator for the impact of media portrayals of mental health stigma on recipients [56].

Recovery narratives are used in successful anti-stigma campaigns [33] [34]. Reduction in self-stigma (typically described by participants as “shame”) was observable as an outcome, providing further validation for this use.

### Strengths and limitations

This paper reports on findings from a substantial qualitative study, drawing on four groups of hard to reach and rarely research participants. A strength of the study design is that it enabled participant reflection [57] on mental states and internal and external events spanning years or decades, and hence supports an understanding of the role of receiving recovery narratives in longer-term recovery processes. A limitation of our study is a lack of respondent validation in relation to the change model produced, and the sample recruited, potentially biased towards participants who are enthusiasts at sharing recovery narratives. In common with prior studies [38–41], participants were only drawn from a single high-income country, and predominantly from two regions in that country (the East Midlands; London). The topic guide asked participants to vocalise a recovery narrative; this may have been constraining for participants with a preference for non-verbal communication. Our analysis method focused on identifying themes that spanned our corpus of transcripts, rather than treating each transcript as an individual case. Participant accounts of recovery narratives were treated as real in our analysis, but recovery narratives might be thought of as retrospective reconstructions, and hence subject to known psychological biases such as selective recall [58].

### Implications

The first framework identifying trans-diagnostic harms from receiving narratives has wide relevance. Any use of mental health recovery narratives should be informed by an understanding that they have the potential to cause pessimism, frustration, a sense of inadequacy and a degree of emotional burden, which may be particularly harmful for those already experiencing distressing mental health difficulties. Training for peer support workers should include approaches to monitoring and addressing these emotional responses when disclosing recover narratives to clients. In line with the biomedical ethical principle of non-maleficence [59], clinicians choosing to integrate recovery narratives into their practice should monitor for negative emotional or cognitive responses. Anti-stigma campaigns that incorporate recovery narratives should evaluate their impact not just in relation to positive changes in community attitudes [60] but also to potential harmful outcomes for people living with mental health difficulties, particularly where recovery narratives are intended for usage in contexts where direct support is not provided [61]. Where collections of recovery narratives are assembled with the intention of enabling use in an unsupported intervention, then intervention developers might design and evaluate tactics for supporting the safety of recipients, such as content warnings, or the selection of “safer” narratives. In a related analysis of how to safely blog about issues such as suicide and self-harm, the anti-stigma campaign Time To Change has identified potentially transferable tactics such as focusing on feeling rather than behaviours, and avoiding explicit discussion of methods [35].

The mechanisms and mediators of impact identified through our analysis have relevance to anyone telling their story of recovery with the intention of helping others, and should therefore shape training that prepares people for employment as peer workers. This might cover techniques to maximise the opportunity for a recipient to feel connected to a peer worker, by incorporating strategies such as talking about relevant but non-visible personal characteristics, or significant recovery events, especially if these have had an influence on how personal challenges were addressed [16]. Empowerment experienced through receiving a recovery narrative may lead to a rapid change in health-related behaviours, and training might seek to empower peers to support recipients through such changes. This has high relevance given that encouraging empowerment is a central feature of peer support [62, 63].

Health service organisations who seek to integrate recovery narratives into their practices should attend to the moderating effect of perceptions of narrative authenticity, especially since a perception that a narrative was being used as a promotional device for a health service offerings was a contributor to perceptions of inauthenticity. Service providers might solicit material which provides a rich, detailed and balanced view of the role of their service in narrator recovery. Providers might adopt a preference to avoid editing, since removing detail apparently irrelevant to the role of a service in recovery may in fact reduce perceptions of authenticity.

Recovery status was repeatedly identified as a characteristic that recipients utilised in comparison, and a greater match appeared to predict impact. Collections of recovery narratives assembled with the intention of promoting recovery might seek to integrate a diverse range of narratives, to maximise the chance of generating useful matches to recipients. A systematic review of research into the characteristics of recovery narratives has provided a nine-dimension framework which might be used to assess diversity of narratives in a collection [1]. Curators might explicitly seek to include narratives that are closer to experiences of distress [8], as these may provide a better match, and hence be more influential for those emerging from distressing periods. The three specific benefits of receiving recorded recovery narratives identified through our analysis confirm the value of collections of recorded recovery narratives. The noted lack of social interaction burden might motivate a specific use as an intervention for people experiencing social anxiety disorders. This is already a motivation for the provision of on-line mental health interventions [64]

Participants interviewed for this study were drawn from four specific groups, and future research might seek to validate this framework with other groups. Some outcomes (especially hope [65]) appear to be more proximal, e.g. immediately and rapidly experiences, whilst others (including stigma reduction and reference shift) may develop more slowly, and hence future work might examine whether there are additional causal relationships between outcomes not represented in our model.

## Supporting information

S1 Text Interview topic guide. **The** final iteration of the interview topic guide used in this study.
(PDF)

S1 Table Refined coding framework produced through thematic analysis of all transcripts. (PDF)

S2 Table Further examples of outcomes coded in transcripts. (PDF)
