# Supplementary material for: The impact of mental health recovery narratives on recipients experiencing mental health problems: Qualitative analysis and change model
Source: PLoS One. 2019 Dec 13;14(12):e0226201. doi: 10.1371/journal.pone.0226201 (PMC6910821; doi:10.1371/journal.pone.0226201)
Supplement: S1 Table — (PDF) [file pone.0226201.s002.pdf]

| Codes describing positive impact of narratives on recipients | | | |
|---|---|---|---|
| **#** | **Code** | **Definition** | **Examples** |
| 1 | Helpful Comparison | Recipient compares Narrator's story to their own and the result is helpful. | Recipient hears a story and uses it to assess their situation, experiences or recovery, with a positive result. These results could include; shift in perspective as Recipient sees that their situation could be 'worse'; Recipient freeing self from assumptions about people with mental health problems, i.e. she is intelligent or capable, and yet has struggled, and so am I; Recipient feeling envious of someone else's recovery and wanting to experience something similar; Recipient compares self to someone who is doing 'badly', and realises they are further along than they thought. |
| 2 | Connectedness | Recipient feels a sense of connection, or reduced isolation, after hearing Narrator's story. | Recipient hears Narrator's story and feels less alone in their experiences, and, perhaps more likely to share their story in the future. Sense of acceptance may contribute to building new, or strengthening existing social relations. |
| 3 | Learning | Recipient learns something helpful, enlightening or interesting from hearing Narrator's story. | Recipient feels that they have learned new skills or understanding by listening to Narrator's story. Could relate to navigating mental health services; learning about self-care, or care of others; learning about political and structural injustices; learning self-acceptance. |
| 4 | Hope | Recipient feels hopeful about the future after hearing Narrator's story. | Recipient hears Narrator's story and feels more positive about the future. May be an individual sense of hope, or a communal one. |
| 5 | Necessary Discomfort | Recipient experiences discomfort from hearing Narrator's story but finds the experience positive overall. | Recipient feels uncomfortable listening to Narrator's story but recognises that it was necessary or important for themselves, or for the Narrator to do so, as something was gained. |
| 6 | Empowerment | Recipient experiences a sense of empowerment from hearing Narrator's story. | Recipient feels encouraged or empowered by hearing Narrator's story, either to continue with their recovery or to tell their own story, in the belief that stories liberate or empower. Belief in the strength of stories and their contagion. |
| 7 | Empathy | Recipient experiences a sense of empathy from hearing Narrator's story, either towards the Narrator, or at a communal level. | Recipient hears Narrator's story and feels empathy towards the individual, or a wider, societal level. Narrator's story could encourage generosity, patience or self-acceptance. |
| 8 | Other positive impact | Any positive impact or benefit for a Recipient which is not coded in above codes. | |

| Codes describing negative impact of narratives on recipients | | | |
|---|---|---|---|
| **#** | **Code** | **Definition** | **Examples** |
| 9 | Unhelpful Comparison | Recipient compares Narrator's story to their own and the result is unhelpful. | Recipient hears a story and uses it to assess their situation, experiences or recovery, with a negative result. These situations could include; Recipient feeling that their story or experiences are not severe enough to warrant assistance or Recipient feeling that their sense of suffering is disproportionate to their experiences and that therefore they have no right to feel as they do. |
| 10 | Despair | Recipient feels despairing about the future after hearing Narrator's story. | Recipient feeling despairing about the future after hearing Narrator's story. |
| 11 | Shame | Recipient experiences feelings of shame after hearing Narrator's story. | Recipient feeling shame or stigma after hearing Narrator's story e.g. if the story involves someone with schizophrenia being assumed to be dangerous, or labelled a 'psycho'. |
| 12 | Unsettled | Recipient experiences discomfort from hearing Narrator's story and finds the experience negative overall. | Recipient feels uncomfortable listening to Narrator's story and believes nothing necessary or important for themselves, or for the Narrator, was gained. |
| 13 | Frustration | Recipient feels frustration from hearing Narrator's story. | Recipient feels frustrated from hearing Narrator's story due to its circularity, tone, or a belief in its inability to create material change. |
| 14 | Harmful | Recipient identifies Narrator's story as having been harmful. | Recipient feels Narrator's story has harmed or damaged them, with nothing positive gained. |
| 15 | Other negative impact | Any negative impact or benefit for a Recipient which is not coded in above codes. | No current examples. |

| Codes describing moderators of impact on recipients | | |
|---|---|---|
| # | Code | Definition |
| 16 | Truth | Impact on Recipient dependant on story's perceived authenticity. |
| 17 | Relatability | Impact on Recipient dependant on story's perceived relatability in terms of Narrator's characteristics or experiences. |
| 18 | Environment | The setting or conditions in which the story is heard. |
| 19 | Timing | The particular point in time in the Recipient’s life at which the story is heard. |
| 20 | Relationship | The relationship between the Recipient and Narrator. |
| 21 | Other moderator | Any other moderator of impact not coded above. |
