# Supplementary material for: The impact of mental health recovery narratives on recipients experiencing mental health problems: Qualitative analysis and change model
Source: PLoS One. 2019 Dec 13;14(12):e0226201. doi: 10.1371/journal.pone.0226201 (PMC6910821; doi:10.1371/journal.pone.0226201)
Supplement: S2 Table — (PDF) [file pone.0226201.s003.pdf]

**Connectedness**
Feeling less alone
I suppose early on was the most helpful for me because um, so maybe hearing Katherine Lucas' story. Because she was one of the first people that I kind of heard about. And it was, it was around helping me feel less isolated. Like I wasn't the only person going through this. So yeah, that would be the main thing that I would think. [A011]<br><br>Well I just think it was you know hearing people's stories and kind of feeling that my God like I am not alone [C006]
Feeling more liked a community member
We did some group work with some women, it was all women, about psychosis. And I started to think, you know, I'm not really on my own, you know. This, it is a very lonely world but I'm not on my own. Hearing about women speaking openly about their experiences of psychosis and how individual and unique it is to them. [B006]<br><br>I think that actually a weird part of my recovery has definitely been connecting with black women, not just about their trauma but I kind of became more connected with listening to black women's stories and looking at black women's' media and on line [B024]
Feeling more connected to specific individuals
I know I connect with people that have had their- that have kind of seen life from a slightly different angle, a bit of a wonky angle. I know I've probably got more in common with them with someone who hasn't, so for me, I've just built up a life of stories since then and try and emulate like, emulate the strongest that I know and just try and be there for the weak as well as- [C005]<br><br>there have been times when I've overshared and then I think oh gosh I wish I hadn't said that thing or in that way and I've learned the hard way about um what to do when that happens and it is that interesting thing and having from my own perspective having been supported by people who have then really openly shared their lived experience being really helpful for me, there is something, it breaks down that judgement, you feel like somebody or that you don't really get it when somebody shares it is that feeling of okay I can connect with you as another human being we have something in common [D014]
**Validation**
Normalising personal experiences
do you know I think in part like it was my middle class friends sharing their traumas so for me to learn like, so one of them had a father that, I don't know how to put it in a way that isn't vile really, well sounds vile, but dropped dead, and another one of them had a dad that jumped in front of a train. And... you know, really bad fucking shit happened and for me learning those things about my friends that I thought I couldn't relate to, finding out that they had had trauma, it just wasn't the trauma that I knew, was really useful. [B024]
Reconceptualising mental health as a collective experiences
sometimes it's nice to know there are other people that are feeling the same as you right now and I think that is like a massive help. Yeah, yeah I think that was like a big help. [D001]

coming across the <anon political network>, volunteering with them for about a year and then realising what I wanted to do with that organisation, I realised has given me the perfect place to actually hear other people and realise, oh right, I'm not that crazy after all, it turns out we are all a bit crazy at some times, so it is really good, yeah, I really value that a lot. [A007]
**Hope and optimism**
Feeling more hopeful about what achievements are possible
When people overcome, when they are at the other side and they are better and they are well, and they have been through it and they know that you do get better, that this is not a permanent situation. It does help, it really helps. You feel, okay I can get there. [A013]<br><br>Yeah that's a good word, inspiring. Yeah. Inspiring. Yes. Inspiring as well to hear when they talk, you feel inspired to think oh you can do that so I can! You know what I mean. [D005]<br><br>Yes. And I take certain things from them. I mean, everybody has a story and everybody has a story and I find that, how they overcome adversity, the strength they find within, resilience, that sort of thing is inspirational to me, yeah. So how they overcome adversity, how they find hope, when there seems like no hope, you know? Yeah, that sort of thing is helpful to me. [B018]<br><br>I think the ones that are successful obviously, the people who have recovered after some horrible things or just a very long fight that people have been in, to see them come out the other side, that- that gives me hope. [C002]<br><br>And then I come across people who through conversation it comes out that they've had some extreme experiences over long periods of time in very early life and you see them walking around and being competent at life, that's inspiring [D008]
Feeling more optimistic about human nature
it's the hope it gives you when someone has gone through something so tough, and it's not that it hasn't affected them. It's not that it's still not with them, but it's that they can move forward and have other positive things happen in their life. And yeah. It's not that they still aren't not there, it's that they can, you know, be part of their personality and see some beauty in that, [D009]
**Empowerment**
Enhanced ability to share personal narratives
So that group had seemed to be so powerful and then what happened was I started to go back regularly and I could bring all the stuff that I couldn't talk about to anyone else I could just bring it all to that group and have no shame or fear of- and I got to know some of the people there and they supported me and they'd say oh do you know what, they wouldn't have the answers all the time but they kind of brought it into sort of a realm of normality almost where it was alright to have it, so what, you know. And that was powerful, that was like somebody had taken a weight off my shoulders that I had been carrying for 6 years [A021]<br><br>when you see somebody talk about thinks, you think 'wow', that person had the, he was brave, step up and talk, that gives you confidence as well. When you hear other people talking about it, when you hear other people talk about it, it gives you, it makes you brave to talk about it. It gives you that, 'OK, I want to talk about it, I want to talk about my experience as well'. [B016]

Enhanced belief that systems can be challenged
When I did mentalisation-based therapy there was a woman who'd had a really horrific experience through the care system and she was taking I think it was the County Council to the Supreme Court and it was for some vicarious liability that she had been placed into a dangerous foster home and had been abused and she wanted to say that the council were liable, that they had a duty of care when they placed her, and they didn't check that it was safe for her. I thought there was no way that she will win, because if she wins that is going to have massive implications, but she did, she won [C019]<br><br>Recently, the activism, I've heard a lot more activism. And people not only have their stories but they're doing something with them, to unite and to change. I'm energised hugely by that. So, yeah. I'm energised hugely by that. [B009]
Enhanced ability to make personal change
But anyway yeah I've heard stories that I've felt myself thinking … that story's worse than mine and you've got through it, so sometimes I'm a bit- even though it was quite horrific those things happening I've heard worse, what I perceive as worse. And so it just puts things in perspective, and opens your eyes again, sometimes uses that wake up nudge thinking yeah I'm not the only one, and that's what I thought when I first walked into the college, oh I'm not the only one. [D006]<br><br>And something about just talking to her that night, I just thought the next day, I just thought I'm not taking the tablet, I'm just not taking it and the next day, I was, I'm not going to take it and I didn't have a clear idea that I was going to stop but I didn't restart and that was several months ago now. [A010]<br><br>the whole reason I was able to first have the confidence to not go back on the medication in the first place was just all the stories I read and there weren't many at the time but Stanislav Groff, his story- his own- His work and his examples and this woman Melissa Gunasala who I found [A010]
**Appreciation**
Enhanced appreciation of positive elements in in the recipient's life
And um, because I now volunteer actually with the <anon network>, which I have done for a few years and I set up um a peer support group in <anon county> a couple of years ago and I suppose now I am hearing from other people that some other people end up sectioned and in hospital and I realise that not everybody that is sectioned would consider it to be a spiritual experience, I always knew it was, uh you know for me it just was as well as I knew that there was mental ill health that went with it, which I guess we would then call psychosis. Um yeah and you know so I suppose from where I sit now I realised 'oh right, I was fortunate and I didn't realise'. [A007]
Enhanced appreciation of the challenges others experience
I have heard stories of other people with abuse issues and that. And it's- I thought yeah mine's bad, but I was pretty humbled. To hear other people's stories, you know. And other people's issues basically. [C012]
**Reference shift**
But during that period I was writing, so I used to keep a journal, I've always kept journals. And so when I got home, I thought, well, if she says that um I don't need any more counselling and I'm better, I want to see where this progression has taken place. So I went back to my journal, and I started to read, and I thought, oh, now I see how depression creeps up on you, now I see how it's so easy to become an alcoholic. And then I said... I carried on, and I

looked for- I started to exercise, and I'd started to clean up my food and I started to do a bit of meditation, I went away with the Buddhist centre, during that- they've had a period of the nine months, or whatever. And I said, oh, wow, this is how you get better then. [A016]<br><br>But anyway yeah I've heard stories that I've felt myself thinking wow, not that you top trump people or anything like that but I think wow that story's worse than mine and you've got through it, so sometimes I'm a bit- even though it was quite horrific those things happening I've heard worse, what I perceive as worse. And so it just puts things in perspective, and opens your eyes again, sometimes uses that wake up nudge thinking yeah I'm not the only one, and that's what I thought when I first walked into the college, oh I'm not the only one. [D006]
**Stigma reduction**
Reduction in shame
and that led me into the um, the mind group, going along to the mind mental health um- cafes where there was a diverse range of people there, some people that were still going through mental health difficulties, some people that had gone through them and now was volunteering- they'd got themselves back to work, and that allowed me to talk to them and use their experiences of what challenges they'd faced from going back to work or you know, what-what I'd sort of been met by them talking about that and things, and one thing I realised was um, that actually much of the mental health stigma existed in me more than what I was being received like on the outside world, so I thought people would think I was nutty but actually the more I spoke about it with other people um, it was- who'd been through similar, or knew people who'd been through similar uh was actually really supportive and um, wanted to help or signpost you to other areas and things.
**Inadequacy**
Others have made a better recovery
Or – or like, “oh my god, that life’s so dreadful and look at them, I would have just killed myself if that had happened to me, and they’re so brave, and that would have destroyed me, and – it can spiral you back down. [D002]
**Disconnection**
From others who have experienced recovery
I've seen people when they were bang at it like I am, then they go and get themselves clean and then it's like a different attitude towards them, they come with this whole different attitude as though they're better than me, you know, just because they've gone and got themselves drug free... and they come- and you should do this, go here, go there, do this and try to push things on to you, because that worked for them. That's fine, if it worked for you it's fine, doesn't say it's going to work for me [C007]
**Pessimism**
About the problems of society
But at the same time, I think we should be trying to create a world that is not, kind of, creating these stories, you know? And to make the world a better place where we don’t have survivors, you know. And I think the emphasis needs to be shifted some more. What is in the ether, what is in the world that is creating these people to have these stories? [B020]
